# *Candida albicans* Dbf4-dependent Cdc7 kinase plays a novel role in the inhibition of hyphal development

**DOI:** 10.1038/srep33716

**Published:** 2016-09-20

**Authors:** Wei-Chung Lai, Tschen-wei Chang, Chang Hao Wu, Shu-Ya Yang, Tai-Lin Lee, Wan Chen Li, Ting Chien, Yu-Che Cheng, Jia-Ching Shieh

**Affiliations:** 1 Department of Biomedical Sciences, Chung Shan Medical University, Taichung City, Taiwan, Republic of China; 2 Department of Molecular Biotechnology, Da-Yah University, Changhua County, Taiwan, Republic of China; 3 Department of Medical Research, Chung Shan Medical University Hospital, Taichung City, Taiwan, Republic of China

## Abstract

*Candida albicans* is an opportunistic human fungal pathogen. The ability to switch among multiple cellular forms is key to its pathogenesis. The Dbf4-dependent protein kinase gene *CDC7* is conserved due to its role in initiating DNA replication. Because a *C. albicans* Cdc7 (*Cacdc7*) homozygous null was not viable, we generated a *C. albicans* strain with a deleted *C. albicans CDC7 (CaCDC7*) allele and an expression-repressible allele. Surprisingly, cells of the strain grew as hyphae under the repressed conditions. The *in vitro* kinase assays confirmed that *Ca*Cdc7 (K232) and *Ca*Cdc7 (T437) are critical for catalytic and phosphoacceptor of activation activity, respectively. *C. albicans* cells formed hyphae when expressing either the catalytically inactive *Ca*Cdc7 (K232R) or the phosphoacceptor-deficient *Ca*Cdc7 (T437A). While *Ca*Cdc7 interacted with *Ca*Dbf4, cells of the strain in which *CaCDC7* was repressed were not rescued by constitutively expressing *C. albicans DBF4* or vice versa. We conclude that *CaDBF4*-dependent *CaCDC7* is an essential gene suppressing the hyphal development.

*Candida albicans* is an opportunistic human fungal pathogen without a complete sexual cycle. The virulence of *C. albicans* stems from its ability to alter morphology from the ellipsoid blastospore to various filamentous forms^1^, although the ability to morphological switch and virulence might be decoupled^2^. Switching among diverse morphological forms is influenced by many environmental factors^3^ and is mediated by several signaling pathways. The mechanism by which hyphal growth is controlled has largely been elucidated and was recently reviewed by Sudbery, P.E.^4^, but the exact roles of many genes known to regulate morphogenesis in polarized growth, cell separation, and the integration of signaling pathways to hyphal growth remain to be determined. Additionally, novel genes involved in morphogenesis remain to be uncovered to understand the overall-control network of the yeast-to-hyphae (YTH) transition. We have identified *S. cerevisiae* homologs of *CDC28*, two G1 cyclins in *C. albicans*^5^, indicating conservation between *C. albicans* and *S. cerevisiae* in the control of the mitotic cell cycle in the G1 phase. However, we and others have shown that *C. albicans CDC4 (CaCDC4*) suppresses filamentation, in contrast to its *S. cerevisiae* counterpart, whose function is required for progression from the G1 to the S phase of the mitotic cell cycle^6^,^7^. We have previously identified several novel Cdc4-associated proteins^8^. Consequently, we investigated other genes required for cells to advance from the G1 to the S phase.

We were particularly interested in the catalytic subunit of the serine/threonine protein kinase encoded by *CDC7* and its regulatory subunit, encoded by *DBF4*, known as Dbf4-dependent Cdc7 kinase (DDK), because they play an essential role in the initiation of DNA replication in *S. cerevisiae*^9^,^10^ and are conserved throughout evolution^11^. In addition to its key role in replication initiation, DDK responds to replication fork stalling^12^,^13^,^14^ and DNA damage^14^,^15^ that maintain genome integrity. Moreover, the roles of DDK are extended to many other areas, including checkpoint control, trans-lesion DNA synthesis, meiosis, chromatin reconstruction, and histone, which have been reviewed recently^16^. Diverse genotoxic insults, including those that block DNA replication, lead to filamentous growth in *S. cerevisiae*^17^,^18^,^19^ and *C. albicans*^20^,^21^,^22^,^23^. The genes involved in DNA replication checkpoints appear to require the induction of filamentous growth^23^. However, how DNA replication stress leads to filamentous growth remains incompletely understood. Moreover, the importance or requirement of some of the key factors, such as Swe1, for DNA replication stress-induced filamentous development does not seem to be conserved between the two yeasts^24^,^25^,^26^,^27^. Until a recent global analysis of *C. albicans* morphology^28^, no reports pointed to a direct involvement of DDK in filamentation.

To verify the role of DDK in *C. albicans*, we have characterized *CaDBF4*^29^ and *C. albicans CDC7 (CaCDC7*). We generated a *C. albicans* strain capable of repressing the expression of *CaCDC7* and examined the cellular morphology when *CaCDC7* is depleted. Strains constitutively expressing the catalytically inactive *Ca*Cdc7 or the phosphoacceptor-deficient *Ca*Cdc7 were generated to verify the requirement of kinase activity for cellular morphology. Additionally, the functional dependency of *Ca*Cdc7 and *Ca*Dbf4 was tested by the yeast two-hybrid assay and by constitutively expressing *Ca*Cdc7 in a *CaDBF*-deletion strain and vice versa.

## Results and Discussion

### *C. albicans CDC7* is a structural homolog of *S. cerevisiae CDC7*

**Figure 1:**
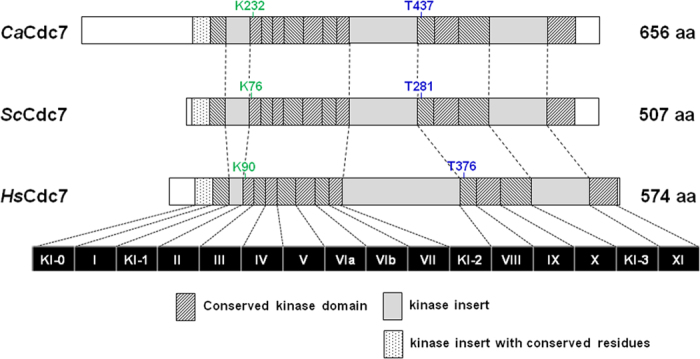
Schematic diagram of the comparison of *C. albicans* Cdc7 (*Ca*Cdc7), *S. cerevisiae* Cdc7 (*Sc*Cdc7), and human Cdc7 (*Hs*Cdc7). The indicated conserved subdomains of the serine/threonine kinases are shown, each of which is specified by a roman numeral (from I to XI). The subdomain VI is further separated into the subdomains VIa and VIb. The kinase insert with conserved residues (KI-0) is shown. The kinase inserts of KI-1, KI-2, and KI-3 are indicated. The positions of the essential lysine residue for catalytic activity of the three Cdc7s, *Ca*Cdc7 (K232), ScCdc7 (K76), and HsCdc7 (K90), are shown. The positions of the phosphoacceptor threonine residue for kinase activation of the three Cdc7s, *Ca*Cdc7 (T437), ScCdc7 (T281), and HsCdc7 (T376), are shown.

By using Blast to compare the Candida Genome Database with the entire sequence of the *S. cerevisiae* Cdc7 protein, a single *CaCDC7* located on chromosome 2 was identified that belongs to Contig19-10183 [70303..72273] orf19.3561, Assembly 19, which has one reading frame of 1971 bp and potentially encodes a 72 kD protein of 657 amino acid residues. To analyze the structure of the protein encoded by *CaCDC7*, the protein sequence derived from the 1971 bp ORF was aligned and compared to other Cdc7 protein sequences across the evolutionary spectrum by ClustalW^30^,^31^. As shown in Supplementary Fig. S1, all twelve conserved serine/threonine protein kinase subdomains as defined by Hanks, S.K., and Quinn, A.M.^32^ can clearly be identified in the protein sequence of *Ca*Cdc7. Moreover, the invariant amino acid residues in the subdomains, which are found throughout protein serine/threonine kinases, are also conserved in *Ca*Cdc7. Based on Supplementary Fig. S1, Fig. 1 was generated as a schematic diagram comparing *C. albicans* Cdc7 (*Ca*Cdc7), *S. cerevisiae* Cdc7 (*Sc*Cdc7), and human Cdc7 (*Hs*Cdc7). As shown in Fig. 1, in domain II of the ATP-binding region, *Ca*Cdc7 contains a lysine at residue 232 (K232), the same as lysine 76 (K76)^33^,^34^ in the budding yeast *Sc*Cdc7 and lysine 90 (K90) in human Cdc7^35^, which is the site required for catalytic activity. In subdomain VIII of the phosphoacceptor region, *Ca*Cdc7 also contains a threonine at residue 437 (T437), equivalent to threonine 281 (T281) in *Sc*Cdc7 and threonine 376 (T376) in humans, which is the phosphorylation site for kinase activation^33^,^34^. Additionally, the spacing between subdomains VII and VIII is worth noting, because the same region in *Sc*Cdc7 is needed for its mitotic function^36^ and is unique among all known kinases^32^. Notably, *Ca*Cdc7 possesses Cdc7 characteristic insertions of KI-0, KI-1, KI-2, and KI-3, and distinct regions located at the N- and C-terminus. Importantly, the C-terminal tail of Cdc7 is known to be essential for interacting with Dbf4^37^. Together with KI-2 and KI-3, such an interaction becomes efficient^38^. Nonetheless, the functional significance of the less-conserved C-terminal tail of *Ca*Cdc7 is unclear. Unique features are also visible in *Ca*Cdc7. The most striking is an extended stretch of approximately 140 amino acid residues, which are quite hydrophobic, at the amino-terminus of *Ca*Cdc7. The region of insertion KI-2 of *Ca*Cdc7 between 355 and 393 is rich in threonine and serine (Supplementary Fig. S1). Nevertheless, the functional significance of these differences is unknown. Despite the differences between *Ca*Cdc7 and its counterparts, particularly *Sc*Cdc7, the overall organization and sequence of *Ca*Cdc7 is very similar to Cdc7. We conclude that *Ca*Cdc7 encodes a serine/threonine kinase with homology both in sequence and organization to Cdc7 across the evolutionary spectrum.

### Construction of the *CDC7* expression-repressible *C. albicans* strain

To establish the function of *CaCDC7* in *C. albicans*, we sought to construct a *Cacdc7* deletion mutant. However, as *CaCDC7* encodes a protein with the structural homologue of known Cdc7 proteins whose function is the initiation of DNA replication, we predicted that the *cdc7* homozygous null mutant is lethal. We addressed this issue by generating a strain with one *CaCDC7* allele deleted and the other under the control of *MET3* promoter (*MET3*p). To delete one *CaCDC7* allele, we used the mini-Ura-blaster approach^39^. We PCR-generated a cassette using the plasmid pDDB57^39^ with the *dpl200* flanked by *URA3* as a template, together with primers having sequences homologous to *URA3*-*dpl200* and the up- and down-stream sequences of *CaCDC7*. We then introduced the cassette into the *C. albicans* auxotrophic strain BWP17 (*ura3 arg4 his1*) to obtain strain *C*a*CDC7*+/*U3*−. Then, *C*a*CDC7*+/*U3*− cells were treated with 5-FOA to obtain strain *C*a*CDC7*+/− whose *URA3* was removed. To obtain a strain capable of conditionally expressing *CaCDC7* under *MET3*p control, we PCR-amplified the partial *CaCDC7* and cloned it into plasmid vector pFA-*HIS1-MET3*p to generate plasmid pFA-*HIS1-MET3*p-p*CaCDC7*. We then linearized the plasmid at a unique restriction site within the partial *CaCDC7*. By introducing the linearized plasmid into strain *C*a*CDC7*+/*U3*−, we obtained strain *C*a*CDC7 M3*/*U3*−. To remove the *URA3* cassette, we treated cells of strain *C*a*CDC7 M3*/*U3*− with 5-FOA to obtain strain *C*a*CDC7 M3*/−. The strains *CaCDC7 M3/U3*−, *C*a*CDC7 M3*/−, *C*a*CDC7*+/*U3*−, and BWP17 were subjected to Southern blotting analysis.

**Figure 2:**
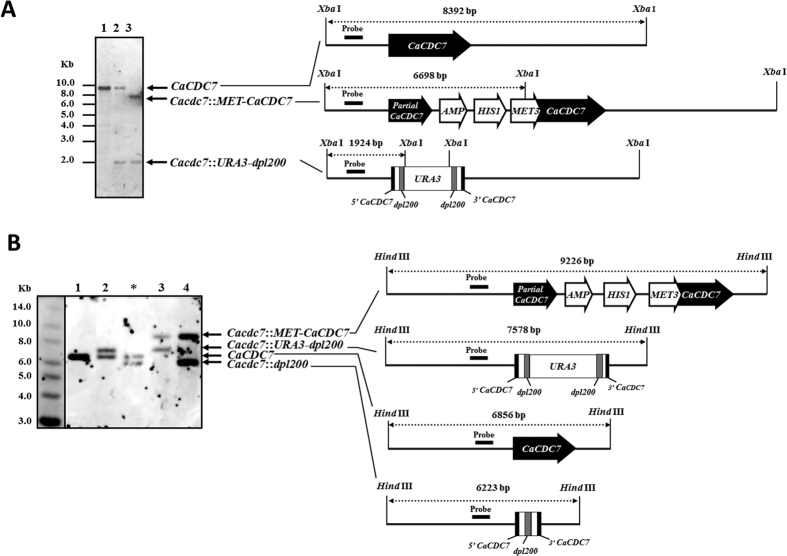
Construction of a *C. albicans* strain capable of repressing the expression of *CaCDC7*. Cells of BWP17 (*CaCDC7*+/+) (#1) were consecutively introduced a cassette of mini-Ura-blaster to obtain *CaCDC7*+/*U3*− (#2) and *MET3*p-driven *CaCDC7* to obtain *CaCDC7 M3/U3*− (#3), after which the *CaCDC7 M3/U3*− was treated with 5-FOA to obtain *CaCDC7 M3/*− (#4). The genomic DNA from cells of each strain was extracted and subjected to either *Xba*I (**A**) or *Hind* III (**B**) digestion before electrophoresis and Southern blotting analysis (the bottom panel). Strain *CaCDC7*+*/*−(*) was made by introducing a cassette of mini-Ura-blaster to BWP17 (*CaCDC7*+/+) to obtain *CaCDC7*+/*U3*−, which was then treated with 5-FOA. The *CaCDC7*+*/*−(*) is used as a control to highlight the relative positions of allele *CaCDC7* and *Cacdc7*::*dpl200*. The relative positions of the probes are shown.

The size of the *Xba*I fragment containing *CaCDC7* shifted from 8392 bp to 1924 bp and from 8392 bp to 6698 bp, respectively, demonstrating that the *CaCDC7* alleles were either integrated with the mini-Ura-blaster cassette or *MET3*p-driven *CaCDC7* (Fig. 2A). The size of the *Hind*III fragment containing *CaCDC7* shifted from 7578 bp to 6223 bp, demonstrating that the *CaCDC7* alleles integrated with the mini-Ura-blaster cassette had lost *URA3* (Fig. 2B). These results indicate that the strain *C*a*CDC7 M3*/− has the expected genome organization of *C. albicans*.

### *CDC7* is an essential gene and depletion of *CDC7* leads to hyphal growth in *C. albicans*

**Figure 3:**
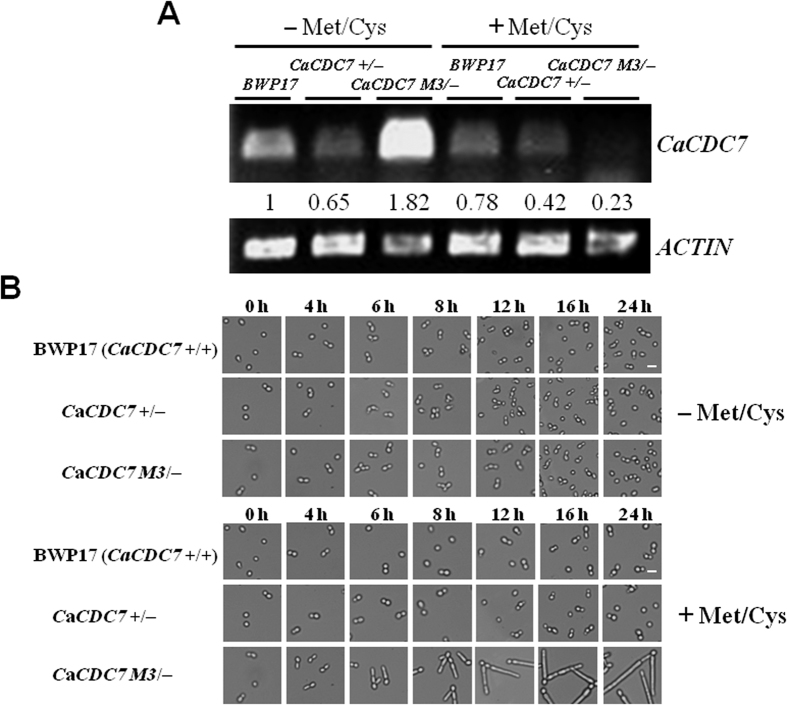
*CaCDC7* suppresses the yeast-to-hypha transition. (**A**) Cells of strains *CaCDC7*+/+ (BWP17), *CaCDC7*+/−, and *CaCDC7 M3/*− were grown in the SD medium with required supplements in the absence (−Met/Cys) or presence (+Met/Cys) of each of 2.5 mM methionine and cysteine at 16 h prior to collection for RT-PCR to verify the repression of *CaCDC7*. The numbers shown are relative fold change in the expression of those strains to BWP17 under the de-repressed condition (−Met/Cys), normalized to *ACTIN* expression. (**B**) The same cultures were grown for the indicated times prior to the assessment of morphological alterations under the microscope. Bars represent 10 μm.

To test the repressibility of *CaCDC7* in the strain *C*a*CDC7 M3*/−, we grew strains *C*a*CDC7 M3*/−, BWP17 (*CaCDC7*+/+), and *CaCDC7*+/− cells in SD medium with or without 2.5 mM methionine/cysteine (Met/Cys) and extracted RNA for RT-PCR analysis. The expression of *CaCDC7* in the strain *C*a*CDC7 M3*/− was significantly reduced under the repressed conditions compared to the de-repressed conditions (Fig. 3A), suggesting that the expression of *CaCDC7* in the strain *C*a*CDC7 M3*/− was solely controlled by *MET3*p and the other allele was deleted.

We next established the requirement for *CaCDC7* in *C. albicans* by growing strains on selective media in the presence or absence of Met/Cys. Strain *C*a*CDC7 M3*/− formed colonies in the presence of Met/Cys with wrinkled surfaces (unpublished data). The results suggested that *CaCDC7* suppresses filamentous development and may not be essential. This result is consistent with our finding that repression of *CaDBF4* expression leads to filamentous growth in *C. albicans*^29^. To confirm the necessity of *CaCDC7* in *C. albicans*, we established a *Cacdc7* homozygous null mutant. However, whereas the *Cacdc7* heterozygous null mutants were generated with ease, no *Cacdc7* homozygous null mutants were obtained. Our result is in agreement with a recent report where the functional copy of the essential *CaCDC7* gene in the heterozygous strain was governed by a tetracycline-repressible promoter to allow functional study^28^. This result is also consistent with the *CaDBF4* data, in which *Cadbf4* homozygous null mutants were unable to survive^29^. These results suggest that *C. albicans CDC7* and its regulator encoded *CaDBF4* gene, like their counterparts across evolutionary spectrum, possesses a conserved role in the initiation of DNA replication. In the *MET3* promoter-driven system, the difference in expression can reach 85-fold between repressed and de-repressed conditions^40^. We reasoned that under the repressed condition, *CaCDC7* was depleted while a limited amount of *Ca*Cdc7 was able to function, although to a lesser extent. While Cdc7 depletion substantially inhibits proliferation in cancer cells^41^,^42^, the depletion of *CaCDC7* appeared to reduce proliferation, as the growth rate of *C*a*CDC7 M3*/− was lower than that of its parental BWP17 when cells were grown in medium with Met/Cys (Supplementary Fig. S2). Taken together, these results suggest that the function of *CaCDC7* is tightly associated to that of *CaDBF4* in *C. albicans* and that the *Ca*Dbf4-dependent *Ca*Cdc7 kinase is essential, as is its counterpart in *S. cerevisiae*^9^,^10^.

To determine the phenotypic consequences, in particular the cellular morphology, of *CaCDC7 M3/*− under the repressed condition, we grew *CaCDC7 M3/*− cells in medium with or without 2.5 mM Met/Cys and examined the phenotypic consequences microscopically. The cells formed germ tubes after 4 h of repression and continued to grow as hyphae from 8 h to 24 h under the repressed condition (Fig. 3B). These observations were comparable to those of cells repressing *CaDBF4* expression^29^, suggesting that *CaCDC7* and *CaDBF4* function as a DDK for the YTH transition and that *CaCDC7* may have an additional role in morphogenesis. To definitely determine that *CaCDC7* is the gene for suppression of the yeast-to-hypha transition, we performed a rescue assay where a constitutive *ACT1* promoter (*ACT1*p)-driven *CaCDC7* was introduced into the *CaCDC7 M3/*− strain. These cells grew in the yeast form even in the presence of Met/Cys (Supplementary Fig. S3), confirming that *CaCDC7* is responsible for the inhibition of hyphal growth in *C. albicans*. Together with the fact that repression of *CaDBF4* expression led to filamentous growth in *C. albicans*^29^, these data suggest that the *CaCDC7* and *CaDBF4* encoded proteins likely act together to perform their function. The hyphal growth during the *CaCDC7* or *CaDBF4* depleted condition may be the consequence of constrained function that exerts as a stress condition. In *S. cerevisiae*, genotoxic stress conditions that reduce DNA synthesis can induce filamentous differentiation through Mec1-Rad53-Swe1-Cdc28-Clb2^17^. However, in *C. albicans*, DNA damage-induced cell cycle delay leading to polarized growth is only partially dependent on Swe1^24^, suggesting that genotoxic stress-induced filamentous growth is not or is only partially mediated by Swe1 in *C. albicans*. However, no evidence indicates that *CDC7* interacts with the above components in *C. albicans*. Hence, whether genotoxic stress-induced filamentous growth is mediated by *CaCDC7* in *C. albicans* remains to be verified.

### The conserved residues are essential for the catalytic activity of *Ca*Cdc7 and the phosphoacceptor of activation

**Figure 4:**
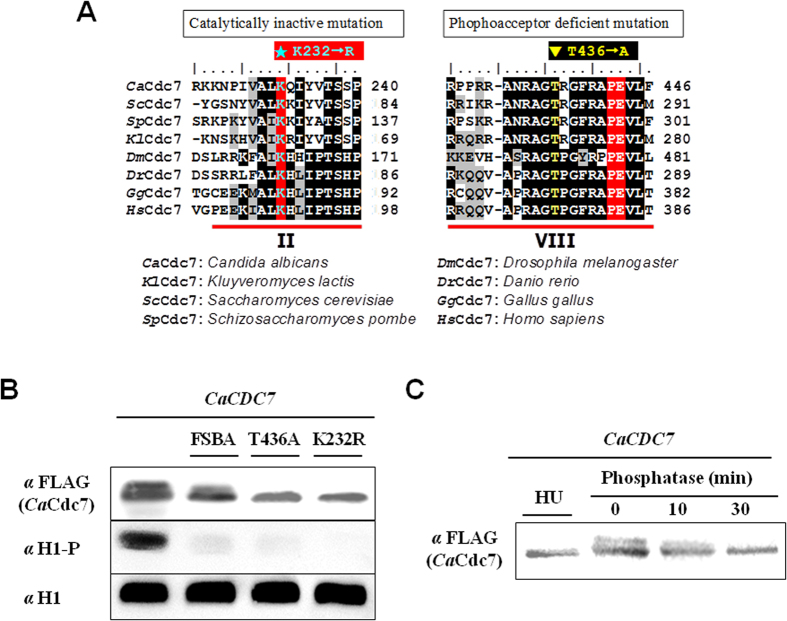
*Ca*Cdc7 possesses conserved sites for kinase catalytic activity and phosphorylation activation. (**A**) The alignment of subdomains II and VII among the homologues of Cdc7, revealing lysine 232 as a conserved residue for catalytic activity and threonine 437 as a conserved phosphoacceptor residue. These were used to generate the catalytically inactive *Ca*Cdc7 (K232R) or the phosphoacceptor-deficient *Ca*Cdc7 (T437A). (**B**) An *in vitro* kinase assay was used to verify that *Ca*Cdc7 (K232) and *Ca*Cdc7 (T437) are required for the catalytic activity and activation by phosphorylation. Cells of strain *CaCDC7* M3/− were transformed with p6HF-*ACT1*p-*Ca*C*DC7*, which is capable of constitutively expressing wild-type *Ca*Cdc7 (*CaCDC7*), catalytically inactive *Ca*Cdc7 (K232R) or the phosphoacceptor-deficient *Ca*Cdc7 (T437A). Cells of each strain were grown in SD medium with the required supplements in the presence (+Met/Cys) of each of 2.5 mM methionine and cysteine for 12 h prior to purification of *Ca*Cdc7 for the *in vitro* kinase assay, followed by western blot analysis with specific antibodies to FLAG (*Ca*Cdc7-6xHisFLAG tagged), histone H1, and phosphorylated histone H1. The purified *Ca*Cdc7 was also subjected to FSBA treatment as described in the Materials and Methods. (**C**) Phosphorylation of *Ca*Cdc7 is activated by hydroxyurea (HU). The cells carrying p6HF-*ACT1*p-*Ca*C*DC7* were grown in same the conditions and media as in (**B**) treated with or without 0.1 mM HU for 1 h. The purified *Ca*Cdc7 from non-HU treated cells was exposed to CIP phosphatase for the indicated times as described in the Materials and Methods.

To verify if the *CaCDC7*-encoded protein product has kinase activity and is capable of being phosphorylated, we created plasmids p6HF-*ACT1*p-*CaCDC7*, p6HF-*ACT1*p-*Ca*C*DC7* (K232R) and p6HF-*ACT1*p-*Ca*C*DC7* (T437A) capable of constitutively expressing either the wild-type *CaCDC7*, the catalytically inactive *Ca*Cdc7 (K232R), or the phosphoacceptor-deficient *Ca*Cdc7 (T437A) (Fig. 4A), each of which was introduced into cells of the strain *CaCDC7 M3*/−. We grew cells of these strains exponentially in the medium with Met/Cys. After harvesting and lysing, each of the wild-type and mutant *Ca*Cdc7s was purified by Ni^2+^-NTA agarose and subjected to an *in vitro* kinase assay. While the wild-type *Ca*Cdc7 was able to phosphorylate histone H1, this phosphorylation was significantly inhibited by FSBA, which blocks the catalytic activity of a kinase (Fig. 4B). This result was consistent with the inability of catalytically inactive *Ca*Cdc7 (K232R) to phosphorylate histone H1 (Fig. 4B). We noted that the FSBA appeared to block autophosphorylation of *Ca*Cdc7, which was indicated by the decrease in the upper band of the *Ca*Cdc7 doublet (Fig. 4B). These results confirmed that *Ca*Cdc7 possesses kinase activity and that the structurally predicted K232 (Fig. 1)^33^,^34^ is indeed the site essential for catalytic activity. Additionally, the phosphoacceptor-deficient *Ca*Cdc7 (T437A) almost lost the ability to phosphorylate histone H1 (Fig. 4B), indicating that the site is the conserved phosphorylation site (Fig. 1)^33^,^34^ that is required for activating kinase activity. The reduction of the upper band of the *Ca*Cdc7 doublet from the purified wild-type *Ca*Cdc7 in a time-dependent manner when treated with phosphatase confirmed that the upper band of the *Ca*Cdc7 doublet is the phosphorylated form of *Ca*Cdc7 (Fig. 4C). A reduction in the phosphorylated form of *Ca*Cdc7 was observed when cells were treated with HU for 1 h before western blot analysis (Fig. 4C). This result is in agreement with the downregulation of *S. cerevisiae* DDK activity mediated by the phosphorylation of Dbf4 through Rad53 in *S. cerevisiae*^12^,^15^,^43^,^44^ and in *Xenopus* egg extracts^45^ under DNA stress, which likely resulted from inhibiting the phosphorylation site of kinase activation.

### The catalytic activity of *Ca*Cdc7 and the phosphorylated activation of *Ca*Cdc7 are required for suppression of hyphal growth in *C. albicans*

**Figure 5:**
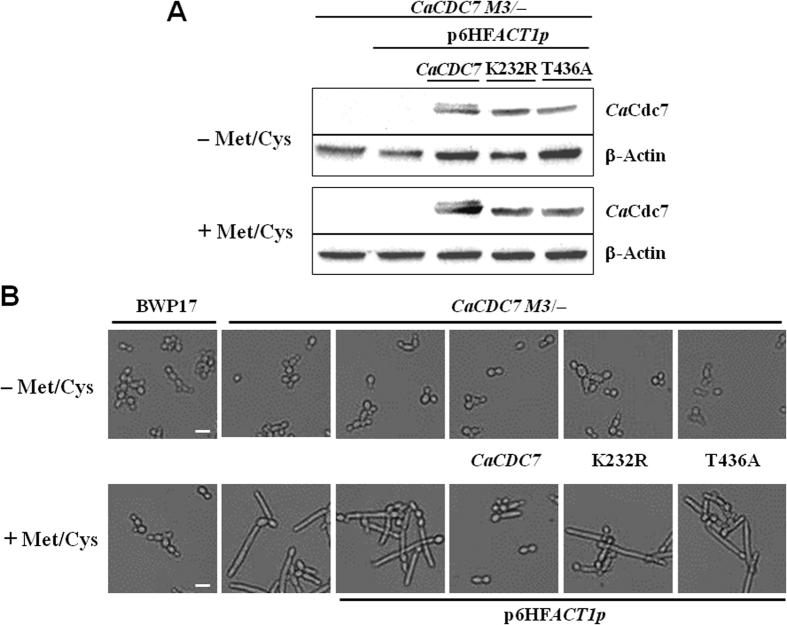
The catalytic activity and phosphorylation of *Ca*Cdc7 are essential for the suppression of the YTH transition in *C. albicans*. (**A**) Cells of strain *CaCDC7* M3/− were transformed with either the empty p6HF-*ACT1*p or p6HF-*ACT1*p-*Ca*C*DC7*, capable of constitutively expressing wild-type *Ca*Cdc7 (*CaCDC7*), catalytically inactive *Ca*Cdc7 (K232R) or the phosphoacceptor-deficient *Ca*Cdc7 (T437A). Cells of each strain, together with the BWP17 from which *CaCDC7* M3/− was derived, were grown in the SD medium with required supplements in the presence (+Met/Cys) or absence (−Met/Cys) of 2.5 mM methionine and cysteine for 12 h at 30 °C prior to the assessment of *ACT*1p-driven protein expression by western blot analysis. (**B**) The same cultures were collected at 12 h for the assessment of morphological alteration under a microscope. Bars represent 10 μm.

To investigate whether the ability of cells to suppress the YTH transition requires kinase activity and phosphorylation of DDK, *CaCDC7 M3*/− cells containing plasmids p6HF-*ACT1*p- *CaCDC7*, p6HF-*ACT1*p-*Ca*C*DC7* (K232R), p6HF-*ACT1*p- *Ca*C*DC7* (T437A) or the empty plasmid p6HF-*ACT1*p were grown in medium with or without Met/Cys. Regardless of the expression of endogenous *CaCDC7* under the control of *MET3*p being repressed or de-repressed, the expression levels of different versions of the *Ca*Cdc7 protein driven under *ACT1*p control were similar (Fig. 5A). It was apparent that cells with repressed *MET3*p-controlled endogenous *CaCDC7* expression but constitutive expression of the *ACT1*p-driven mutants *Ca*Cdc7 (K232R) or *Ca*Cdc7 (T437A) grew as the hyphal form (Fig. 5B), which is in contrast to those expressing *ACT1*p-driven wild type *Ca*Cdc7, which remained as yeast cells. These results suggest that the catalytic activity of *Ca*Cdc7 and the phosphorylation of *Ca*Cdc7 are required for the function of *Ca*Cdc7 on the suppression of the YTH transition.

By examining cells of the strains repressing the expression of *CaCDC7* in a longer time, we confirmed that the mutant strains were able to proliferate, though in a reduced rate as verified by the growth curve (Supplementary Fig. S2). The constitutive expression of *CaCDC7* but none of the two *CaCDC7* mutants allows recovery of this growth defect (Supplementary Fig. S2). These data indicate that cells depleted with *CaCDC7* or expressing the defective *Ca*Cdc7 mutants delay the cell cycle progression. We note that *cdc7* conditional mutants of *S. cerevisiae* show a dumbbell shape^46^, unlike polarized growth of *C. albicans* expressing the *CDC7* mutations. It is known that pseudohyphae and true hyphae emerge in response to cell-cycle arrest in *C. albicans*^47^. Hence, the *C. albicans* expressing the mutant *CDC7* may delay the cell cycle at the S phase. However, the cells were still able to grow, and the extension of hyphal development was more prominent at a later time point of 32 h (Supplementary Fig. S4). Moreover, because the cell cycle stage of cells repressing with *CaCDC7* was different, the budded daughter cells, like the mother cells, switch to the hyphal mode of growth can grow as extended hyphal form (Fig. 5). These results suggest that cells depleted with *CaCDC7* or expressing the defective *CaCDC7* mutants delay in the cell cycle, which accompanies the reduced rate of hyphal extension. To assess further if *CaCDC7* is required for YTH, cells of the strains, together with BWP17 were subjected to grow in 37 °C, a condition known to induce hyphal growth. As shown in the Supplementary Fig. S5A, however, the hyphal formation appeared in the *CaCDC7*-repressede but not the *CaCDC7*-derepressed condition at 37 °C, suggesting that the initiation and continuation of filamentation are dependent on *Ca*Cdc7 in *C. albicans*. Importantly, unlike the wild-type strain SC5314, BWP17was unable to induce hyphal development but pseudohyphae-like type when cultured in the SD minimum medium at 37 °C (Supplementary Fig. S5B).

We are not aware of any Cdc7 homologs across the evolutionary spectrum that are involved in morphogenesis until the latest report. Based on the GRACE strain collection^48^,^49^, the report identified over a hundred strains as negative regulators of filamentation. The expression of *CaCDC7* of the GRACE strain was controlled by the *tet*-operator; hence, cells formed filaments under the repressed condition^28^. However, the kinase activity of *Ca*Cdc7 and its activation by phosphorylation has not been determined.

### *CDC7* and *DBF4* are interdependent for the function of morphological control in *C. albicans*

**Figure 6:**
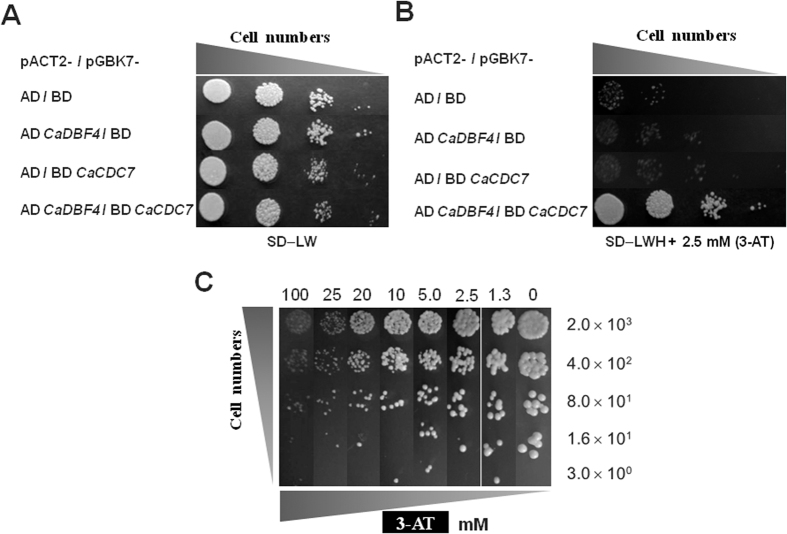
Specific interaction between *CaCDC7* and *CaDBF4* verified by a yeast two-hybrid assay. (**A**) Verification of diploid *S. cerevisiae* cells carrying the plasmids required for the test. The plasmids pACT2 and pACT2-*CaDBF4* (capable of expressing Gal4 activation domain fused with *Ca*Dbf4) were transformed into AH109, from which AD and AD *CaDBF4* were obtained, respectively. The plasmids pGBKT7 and pGBKT7-*CaCDC7* (capable of expressing Gal4 DNA biding domain fused with *Ca*Cdc7) were transformed into Y187, from which BD and BD *CaCDC7* were obtained, respectively. AH109 and Y187 derivatives were mated to obtain diploid *S. cerevisiae* cells of AD/BD, AD *CaDBF4*/BD, AD/BD *CaCDC7*, and *AD CaDBF4*/*BD CaCDC7*. Ten-fold serially diluted *S. cerevisiae* diploid cells (10^4^∼10^1^) were grown on semi-solid agar plates with selective minimum media lacking leucine and tryptophan (SD−LW). (**B**) Verification of the interaction between CaCdc7 and CaDbf4. Ten-fold serially diluted *S. cerevisiae* diploid cells (10^4^∼10^1^), the same as in (**A**) were grown on semi-solid agar plates with selective minimum media lacking leucine, tryptophan, and histidine, supplemented with 2.5 mM 3-AT, the antagonist of the *HIS3* gene product (SD−LWH+2.5 mM 3-AT). (**C**) Strong interaction occurs between *Ca*Cdc7 and *Ca*Dbf4. Serially diluted *S. cerevisiae* diploid cells of *AD CaDBF4*/*BD CaCDC7* were grown on semi-solid agar plates with selective media lacking leucine, tryptophan, and histidine but with the indicated concentration of 3-amino-triazole (3-AT). The cells were able to proliferate normally at the concentration of 20 mM due to a relatively strong interaction between *Ca*Cdc7 and *Ca*Dbf4.

The protein products encoded by *CDC7* and *DBF4* function together as a DDK for the initiation of DNA replication^10^. Although our study suggests that DDK in *C. albicans* plays a significant role in morphogenesis, we wondered whether the presence of *C. albicans* DDK functions in hypha-suppression in *C. albicans.* The definitive determination of how *Ca*Cdc7 and *Ca*Dbf4 constitute DDK required verification of their direct interaction. To do so, we adopted the yeast two-hybrid assay. The diploid *S. cerevisiae* cells were able to grow on media lacking leucine and tryptophan, indicating the presence of both plasmids based on the Gal4 DNA binding domain with the *TRP1* selection marker and the Gal4 activation domain with the *LEU2* selection marker (Fig. 6A). However, only those cells expressing *Ca*Cdc7 fused with the Gal4 DNA binding domain and *Ca*Dbf4 fused with the Gal4 activation domain concurrently were able to grow on the plate without histidine (Fig. 6B). These results suggest that *Ca*Cdc7 and *Ca*Dbf4 can associate physically. Moreover, diploid *S. cerevisiae* cells capable of simultaneously expressing *Ca*Cdc7 fused with the Gal4 DNA binding domain and *Ca*Dbf4 fused with the Gal4 activation domain were able to grow on the plate without histidine, even in the presence of 20 mM 3-amino-1,2,4-triazole (3-AT), the inhibitor of imidazole glycerol-phosphate dehydratase encoded by the reporter gene *HIS3*. These results indicate that the interaction between *Ca*Cdc7 and *Ca*Dbf4 is relatively stable and thus likely to be genuine (Fig. 6C).

**Figure 7:**
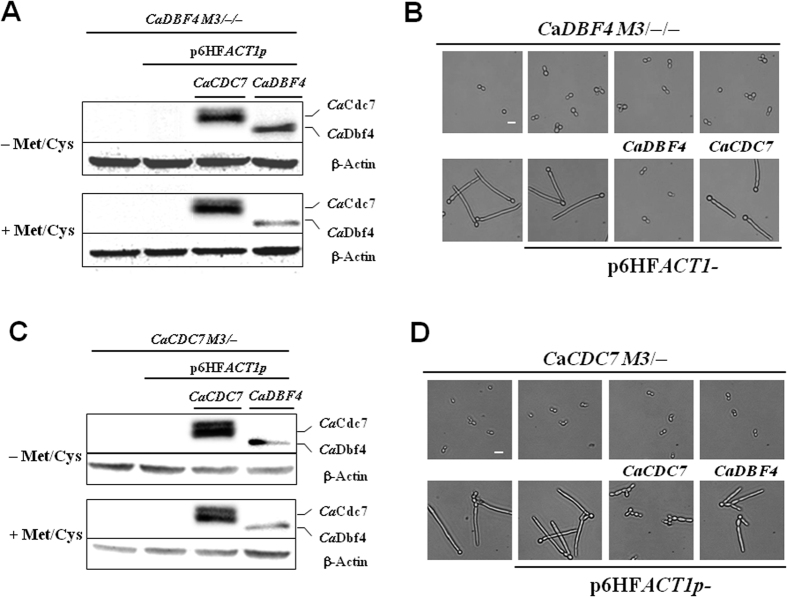
Constitutive expression of *CaCDC7* cannot release the expression of repressed *CaDBF4,* nor can *CaDBF4* release expression-repressed *CaCDC7* on filamentation in *C. albicans*. Cells of strain *CaCDC7* M3/− and those with either the empty p6HF-*ACT1*p or p6HF-*ACT1*p-*CaDBF4* and p6HF-*ACT1*p-*Ca*C*DC7*, which are capable of constitutively expressing *Ca*Dbf4 and *Ca*Cdc7, respectively, were grown in SD medium with the required supplements in the presence (+Met/Cys) or absence (−Met/Cys) of 2.5 mM methionine and cysteine for 12 h prior to the assessment of protein expression by western blotting analysis (**A**) and assessment of the morphological consequences microscopically (**B**). Cells of strain *CaDBF4* M3/−/− (ref) and those with either the empty p6HF-*ACT1*p or p6HF-*ACT1*p-*Ca*C*DC7* and p6HF-*ACT1*p-*CaDBF4,* which are capable of constitutively expressing wild-type *Ca*Cdc7 and *Ca*Dbf4, respectively, were grown in the same conditions as above prior to the assessment of protein expression (**C**) and microscopic assessment of the morphological consequences (**D**). β-Actin was used as a loading control in the analyses of protein expression by western blotting. Bars represent 10 μm in the microscopic observations.

To assess the functional dependency between *CaCDC7* and *CaDBF4*, we generated plasmids p6HF-*ACT1*p-*Ca*C*DC7* and p6HF-*ACT1*p-*CaDBF4* that are capable of constitutively expressing either *CaCDC7* or *CaDBF4,* and introduced them into *CaDBF4 M3*/−/−29 and *CaCDC7 M3*/−, respectively. The ability of a DDK gene that can be constitutively expressed to suppress the loss of the other DDK gene that is repressed in the presence of Met/Cys was assessed. Constitutive expression of *CaCDC7* (Fig. 7A) did not suppress the loss of *CaDBF4* (Fig. 7B), suggesting that the function of *CaDBF4* requires the presence of *CaCDC7*. Similarly, constitutive expression of *CaDBF4* (Fig. 7C) was unable to suppress the loss of *CaCDC7* (Fig. 7D). The epistasis analyses and yeast two-hybrid assay results confirm that *CaCDC7* and *CaDBF4* encode proteins that form functional DDK and are functionally interdependent. Additionally, we introduced a Tet-on expression system^50^ into BWP17 where *CaCDC7* or *CaDBF4* were massively overproduced in the presence of doxycycline under serum-induced hyphal growth conditions. Cells under such conditions grew as hyphae (Supplemmentary Fig. S6), suggesting that either DDK control of the yeast-to-hypha transition is not via the serum-induced signaling pathway, but instead through other pathways, such as DNA replication stress, or that the blockage of serum-induced filamentous growth could be bypassed by other factors. It is equally possible that DDK control of the yeast-to-hypha transition constitutes a novel pathway.

## Conclusion

In this paper, we describe the characterization of the *CaCDC7* gene. To our surprise, we found that *C. albican*s *CDC7* plays a role as a negative regulator of the YTH transition. Additionally, kinase activity is required for the function of *Ca*Cdc7 and the presence of other kinases for the function of *Ca*Cdc7. Moreover, the function of *Ca*Cdc7 is dependent on *Ca*Dbf4 for suppression of the hyphal mode of growth. To the best of our knowledge, no known role for Dbf4-dependent Cdc7 homologs across the evolutionary spectrum relevant to morphogenesis has been identified. The uncovering of *Ca*Cdc7 function in morphogenesis may be of evolutionary significance in that essential elements in the cell cycle have evolved in the direction of morphogenesis for adapting the host-pathogen interaction. It would be crucial to elucidate the regulation of DDK in the network controlling morphogenesis. Elucidating the regulation of DDK in the system that controls morphogenesis should lend insight into understanding why DDK is involved in morphogenesis.

## Methods

### General manipulation, media and growth conditions

The *E. coli* strain DH5α was used for routine manipulation of the plasmids, and all *C. albicans* strains (Table 1) were derived from the auxotrophic strain BWP17 (*arg4/arg4 his1/his1 ura3/ura3*)^51^. The wild-type SC5314 strain^52^ was also used. The media usage and routine growth conditions of the strains of *E. coli* and *C. albicans* were as previously described^53^. *E. coli* strain DH5α was transformed with plasmid DNA by CaCl_2_ as described^54^ or by electroporation^55^. *Candida albicans* strains were transformed by the LiAc/PEG/ssDNA method^56^ or electroporation^57^.

### Strain construction

The nucleotide sequence of *C. albicans* was obtained from the *Candida* Genome Database (http://www.candidagenome.org/). The *CaCDC7* gene including regions flanking the protein coding sequence was PCR-generated with the primers CaCDC7-xba-F and CaCDC7-xba-R (Table 2) and genomic DNA extracted from *C. albicans* strain BWP17. The *CaCDC7* gene was cloned into the plasmid vector pBluescript II (+) to obtain the plasmid pBII-*CaCDC7. CaCDC7* was deleted in the *C. albicans* auxotrophic strain BWP17 with the mini-Ura-blaster cassette *dpl200*-*URA3*-*dpl200* derived from pDDB57^39^. Briefly, the mini-Ura-blaster cassette flanked by 60 bp of upstream and downstream homology to the *CaCDC7* open reading frame was amplified by PCR with the template pBII-*CaCDC7* and a pair of primers, CaCDC7-Spe-F and CaCDC7-Spe-R (Table 2). The mini-Ura-blaster cassette flanked by the short homology regions of *CaCDC7* was then transformed into BWP17 cells and the uridine prototrophic strains (Ura^+^) *CaCDC7*+/*U3*− (Table 1) containing the heterozygous deletion of *CaCDC7* were selected for. To allow *CaCDC7* expression under the control of *MET3*p, a partial *CaCDC7* ORF (1–884 bp) flanked by an *Spe*I site was PCR generated with the template pBII-*CaCDC7* and a pair of primers, CaCDC7-spe-F and CaCDC7-spe-R (Table 2). The PCR product was cloned into pFA-*HIS1*-*MET3*p^40^ at the *Spe*I site and the plasmid pFA-*HIS1*-*MET3*p-*CaCDC7* was obtained. Plasmid pFA-*HIS1*-*MET3*p-*CaCDC7* was linearized by digesting with a unique *EcoR*I on partial *CaCDC7* and was transformed into *CaCDC7*+/*U3*− to generate the His^+^ prototrophic strain *CaCDC7 M3*/*U3*−. To remove the *URA3* marker, the cells of *CaCDC7 M3*/*U3*− were then treated with 1 mg/ml 5-FOA to generate *CaCDC7 M3*/− (Table 1). To repress the *CaCDC7* expression that is controlled by *MET3*p, strains were grown in SD medium or on a plate with 2.5 mM Met/Cys, which turns off the expression of *MET3*p-driven downstream genes^40^. To allow constitutive expression of *CaCDC7* in *C. albicans* cells, the protein coding sequence of *CaCDC7* was cloned from the plasmid pBII-*CaCDC7* with a pair of primers, CaCDC7-Sph-F and CaCDC7-Sph-R (Table 2), and introduced into plasmid p6HF-*ACT1*p^58^ to generate p6HF-*ACT1*p-*CaCDC7*. The p6HF-*ACT1*p-*CaCDC7* and the empty plasmid p6HF-*ACT1*p were linearized with *Nco*I and introduced into *CaCDC7 M3*/−. These cells were selected for Ura^+^ transformants that targeted and integrated at the *RP10* locus to generate *CaCDC7M3*/−|*CaCDC7* and *CaCDC7M3*/−|p6HF-*ACT1*p (Table 2), respectively. The linearized plasmids were also introduced into *CaDBF4 M3*/−/−29 to obtain *CaDBF4 M3*/−/−|*CaCDC7* and *CaDBF4 M3*/−/−|p6HF-*ACT1*p (Table 2), respectively. In addition, the linearized plasmid p6HF-*ACT1*p-*CaDBF4* (manuscript submitted) was introduced into *CaCDC7 M3*/− to generate *CaCDC7M3*/−|*CaDBF4.*

### Site-directed mutagenesis

The QuickChange^TM^ Site-Directed Mutagenesis Kit (Stratagene, San Diego, CA) was used as instructed by the manufacturer to introduce mutations in *CaCDC7* using the plasmid p6HF-*ACT1*p-*CaCDC7*. Following site-directed mutagenesis with two pairs of primers, CaCDC7-K232R-F/CaCDC7-K232R-R and CaCDC7-T436A-F/ CaCDC7-T436A-R (Table 2), the plasmids p6HF–*ACT1*p-*Ca*C*DC7* (K232R) and p6HF-*ACT1*p- *Ca*C*DC7* (T437A) were generated, respectively. Each of the plasmids (p6HF–*ACT1*p-*Ca*C*DC7* (K232R), p6HF-*ACT1*p–*Ca*C*DC7* (T437A) was *Nco*I-linearized and introduced into *C. albicans* strain *CaCDC7 M3*/− and targeted and integrated at the *RP10* locus to generate *CaCDC7M3*/−|K232R and *CaCDC7M3*/−|T437A, respectively.

### Yeast two-hybrid assay

The yeast two-hybrid assay was used. Plasmid constructs capable of expressing the Gal4 activation domain fused N-terminally to *Ca*Dbf4 (pACT2-*CaDBF4*) or the Gal4 DNA binding domain fused N-terminally to *Ca*Cdc7 (pGBKT7-*CaCDC7*) were made using genomic DNA extracted from BWP17 with two pairs of oligonucleotides, CaDBF4-XmaI-F and CaDBF4-XhoI-R (Table 2) and CaCDC7-NcoI(hybrid)-F and CaCDC7-PstI(hybrid)-R (Table 2), respectively. The plasmids pGBKT7-*CaCDC7* and pACT2-*CaDBF4* were introduced into the haploid *S. cerevisiae* strain Y187^59^ and AH109, a derivative of strain PJ69-2A^60^ resulting from the introduction of the lacZ reporter gene into PJ69-2A (Table 1), respectively, with opposite mating types and with the reporter systems *HIS3*, *ADE2*, and *LacZ.* These strains were used to determine the activation of the system and the interaction between *Ca*Cdc7 and *Ca*Dbf4 upon fusion to become diploid.

### Isolation of genomic DNA and Southern blotting

Genomic DNA from the *C. albicans* strains was isolated by the MasterPure^TM^ Yeast DNA Purification Kit (EPICENTRE). Southern blotting was performed according to standard protocols with the aid of the Rapid Downward Transfer System (TURBOBLOTTER^TM^) using 10 μg of the restriction enzyme-digested genomic DNA. The probe was generated by the PCR DIG probe synthesis kit (Roche) with a genomic DNA template extracted from BWP17 and a pair of primers, CaCDC7-Probe-F and CaCDC7-Probe-R (Table 2). The DNA on the blot was hybridized with the probe using DIG Easy Hyb (Roche). To reveal the gene deletion, the DIG Luminescent Detection Kit (Roche) was used after hybridization, and the blot was exposed to X-ray film for up to 24 h as appropriate.

### RT-PCR analysis

Cells were grown to mid-log phase and total RNA was extracted using the MasterPure^TM^ Yeast RNA Purification Kit (EPICENTRE) following the manufacturer’s instructions. Then, 5 μg of total RNA was used to generate cDNA by using the SuperScript III Reverse Transcriptase Kit (Invitrogen) following the manufacturer’s instructions. The cDNA was then subjected to PCR with a pair of *CaCDC7*-specific primers, CaCDC7-internal-F-2 and CaCDC7-BspEI-R (Table 2), targeted downstream of the coding sequence. A 366 bp product was generated.

### Immunoblot analysis

The total protein was extracted from cultured cells as previously described^61^. The protein was partially purified from cells bearing the p6HF-*ACT1*p plasmid with the open reading frame of the gene integrated at *RP10* capable of generating a tagged (6 × His and FLAG) protein using Ni^2+^-NTA-agarose beads (Qiagen Inc.) essentially as previously described^61^. Precipitated proteins were resolved by 10% SDS-PAGE and transferred electrophoretically to PVDF membranes (PerkinElmer, Boston, USA) and probed with a polyclonal antibody to FLAG (Sigma) in a 1:2000 dilution and visualized using the SuperSignal West Pico Chemiluminescent Substrate Kit (PIERCE). The detected proteins were recorded with the Luminescent Image Analyzer (FUJIFILM LAS-1000) and analyzed by ImageGauge 3.46 and L Process v 1.96 (FUJIFILM).

### *In vitro* kinase assay

The *in vitro* kinase assays using *Ca*Cdc7*-*6xHisFlAG and purified bovine histone H1 (Upstate) as substrates were performed using a non-radioactive assay essentially as previously described^62^. Briefly, the Ni^2+^-NTA-agarose bead (Qiagen Inc.)-purified *Ca*Cdc7 was washed three times with kinase reaction buffer [25 mM Tris pH 7.5, 5 mM β-glycerophosphate, 2 mM DTT, 0.1 mM Na_3_VO_4_, 10 mM MgCl_2_ and a serine/threonine phosphatase inhibitor cocktail (Calbiochem)] and used in the kinase reaction with 5 μg histone H1 as substrate in the presence of 200 μM ATP in a 20 μl kinase assay buffer. The kinase reaction was incubated at 30 °C for 45 minutes, and the end products were resolved by 12% SDS-PAGE and detected by immunoblotting using anti-Histone H1 (H11-4, Sigma; 1:500) and anti-phospho-Histone H1 (12D11, Millipore; 1:1000 monoclonal antibodies) according to the manufacturer’s instructions.

### Chemical treatment

Phosphatase treatment was performed with calf intestinal alkaline phosphatase (CIP) (NEB). Briefly, the Ni^2+^-NTA-agarose bead (Qiagen Inc.)-purified *Ca*Cdc7 was washed three times with CIP buffer (1XNE Buffer 3) and resuspended in 20 μl CIP buffer before the addition of 20 units of CIP and incubation at 37 °C for the required period of time. The CIP was inactivated by heating to 75 °C for 10 minutes in the presence of 5 mM EDTA. Irreversible protein kinase inhibitor treatment was performed with 5′-fluorosulfonylbenzoyl-5′-adenosine (FSBA), essentially as previously described^63^. Briefly, the Ni^2+^-NTA- agarose bead-purified *Ca*Cdc7 was subjected to three successive treatments by 1 mM FSBA (Sigma) at 30 °C for 15 min in kinase buffer without DTT. The product was then washed three times with kinase buffer to eliminate the FSBA before performing the *in vitro* kinase assay. Hydroxyurea (HU) was used to block DNA replication, which is known to attenuate Cdc7 kinase activity^15^. Cells in culture were treated with 0.1 M HU before the Ni^2+^-NTA- agarose bead purification of *Ca*Cdc7.

### Cellular image observation and recording

Cells in liquid culture were visualized and recorded with a Nikon 50i microscope at 400x magnification. Colonies were photographed with a MEIJI stereoscopic microscope EMZ5 at 40x magnification.

## Additional Information

**How to cite this article**: Lai, W.-C. *et al.* *Candida albicans* Dbf4-dependent Cdc7 kinase plays a novel role in the inhibition of hyphal development. *Sci. Rep.* **6**, 33716; doi: 10.1038/srep33716 (2016).

## Supplementary Material

Supplementary Information

## Figures and Tables

**Table 1:** Strains used in this study.

Name of the strain	Parental strain	Genotype	Source
*Candida albicans*			
BWP17		*ura3*::imm434*/ura3*::imm434 *iro1/iro1*::imm434 *his1*::*hisG /his1*::*hisG arg4*::*hisG/arg4*::*hisG*	Reference^49^
*CaCDC7*+/*U3*−	BWP17	*CaCDC7/Cacdc7*::*URA3-dpl200*	This study
*CaCDC7*+/−	*CaCDC7*+/*U3*−	*CaCDC7/Cacdc7*::*dpl200*	This study
*CaCDC7 M3*/*U3*−	*CaCDC7*+/*U3*−	*Cacdc7*:: *URA3-dpl200*/*Cacdc7*::pFA-*HIS1*-*MET3*p-*CaCDC7*	This study
*CaCDC7*+/−	*CaCDC7*+/*U3*−	*CaCDC7/Cacdc7*::*dpl200*	This study
*CaCDC7 M3*/−	*CaCDC7*+/−	*Cacdc7*::*dpl200*/*Cacdc7*::pFA-*HIS1*-*MET3*p-*CaCDC7*	This study
*CaCDC7 M3*/− |p6HF-*ACT1*p	*CaCDC7 M3*/−	*Cacdc7*::*dpl200*/*Cacdc7*::pFA-*HIS1*-*MET3*p-*CaCDC7RPS1/rps1::*p6HF- *ACT1*p	This study
*CaCDC7 M3*/− |*CaCDC7*	*CaCDC7 M3*/−	*Cacdc7*::*dpl200*/*Cacdc7*::pFA-*HIS1*-*MET3*p-*CaCDC7RPS1/rps1::*p6HF-*ACT1*p-*CaCDC7*	This study
*CaCDC7 M3*/− |K232R	*CaCDC7 M3*/−	*Cacdc7*::*dpl200*/*Cacdc7*::pFA-*HIS1*-*MET3*p-*CaCDC7RPS1/rps1::*p6HF-*ACT1*p-*CaCDC7*(K232R)	This study
*CaCDC7 M3*/− |T437A	*CaCDC7 M3*/−	*Cacdc7*::*dpl200*/*Cacdc7*::pFA-*HIS1*-*MET3*p-*CaCDC7RPS1/rps1::*p6HF-*ACT1*p-*CaCDC7*(T437A)	This study
*CaCDC7 M3*/− |*CaDBF4*	*CaCDC7 M3*/−	*Cacdc7*::*dpl200*/*Cacdc7*::pFA-*HIS1*-*MET3*p-*CaCDC7RPS1/rps1::*p6HF-*ACT1*p-*CaDBF4*	This study
*CaDBF4 M3*/−/− |*CaCDC7*	*CaDBF4 M3*/−/−	*Cadbf4*::*dpl200*/*Cadbf4::ARG4/Cacdc7*::pFA-*HIS1*-*MET3*p-*CaDBF4RPS1/rps1::*p6HF-*ACT1*p-*CaCDC7*	This study
*CaDBF4 M3*/−/− |p6HF-*ACT1*p	*CaDBF4 M3*/−/−	*Cadbf4*::*dpl200*/*Cadbf4::ARG4/Cacdc7*::pFA-*HIS1*-*MET3*p-*CaDBF4RPS1/rps1::*p6HF-*ACT1*p	Reference^29^
***Saccharomyces cerevisiae***			
Y187		*MATα, ura3-52, his3-200, ade2-101, trp1-901, leu2-3, 112, gal4Δ, met*−*, gal80Δ, MEL1, URA3*::*GAL1UAS -GAL1TATA-lacZ*	Reference^56^
AH109		*MATa, trp1-901, leu2-3, 112, ura3-52, his3-200, gal4∆, gal80∆, LYS2::GAL1UAS-GAL1TATA-HIS3, GAL2UAS-GAL2TATA-ADE2, URA3*::*MEL1UAS-MEL1 TATA-lacZ*	Reference^57^ as described in the Materials and Methods

**Table 2:** Synthetic oligonucleotide primers used in this study.

Name	Sequence(5′→3′)^1^,^2^
CaCDC7-xba-F	GAGTCTAGACCCTAACGACATTTGCTGAA
CaCDC7-xba-R	CCCTCTAGAAACGCAAACACAAGAGCAA
CaCDC7-URA3-F	CTTATAAATTTGTGATTAAAAATCTGAACTTTTGAGGTTAGTCTCTTTTTTATTTTTTAATTTTCCCAGTCACGACGTTG
CaCDC7-URA3-R	GTTATTTTATTTCTGTATGTGTAATTAAATTTATATCAAATTATAAACTATATAAACTATTTGTTTGTGGAATTGTGAGCGG
Check URA3-F	CATTGTTGGTAGAGGATTGT
CaCDC7-spe-F	GCCACTAGTATGCAAGAAGTTTTGTTTAC
CaCDC7-spe-R	GCCACTAGTCCTTTAACGGGTAAGTCA
CaCDC7-Sph-F	CATAAGCATGCATGCAAGAAGTTTTGTTTAC
CaCDC7-Sph-R	GCCATGTGCATGCAGATAAAATTACTTCATCTTC
CaCDC7-probe-F	CTTGAGGATGTAATCCATTAG
CaCDC7-probe-R	CAAGATTGTTCAACCCTCTC
CaCDC7-intermnal-F-2	GGATGTTTCCCATCTGATAG
CaCDC7-BspEI-R	GCCTCCGGACTAAGATAAAATTACTTCATC
CaACT1-F	ACGGTGAAGTTGCTGCTTTA
CaACT1-R	GCATTTCTTGTTCGAAATCC-3′
CaCDC7-K232R-F	CCTATAGTTGCTTTAAGACAAATCTATGTCACGTCTTCCCCC
CaCDC7-K232R-R	GGGGGAAGACGTGACATAGATTTGTCTTAAAGCAACTATAGG
CaCDC7-T436A-F	GCCAATAGGGCTGGAGCTAGAGGTTTTAGAGC
CaCDC7-T436A-R	GCTCTAAAACCTCTAGCTCCAGCCCTATTGGC
CaCDC7-NcoI(hybrid)-F	CATGCCATGGGCATGCAAGAAGTTTTGTTTACATA
CaCDC7-PstI(hybrid)-R	AAAACTGCAGCTAAGATAAAATTACTTCATCTTC
CaDBF4-XmaI-F	TCCCCCCGGG GATGTCGAAAGTGGAAGAGCA
CaDBF4-XhoI-R	GGC*C*TCGAGCTATACATAATCGCCGTTTG

^1^Restriction enzyme sites are shaded in grey.

^2^The nucleotides to be mutated are framed.
