# Supplementary material for: Candida albicans Dbf4-dependent Cdc7 kinase plays a novel role in the inhibition of hyphal development
Source: Sci Rep. 2016 Sep 20;6:33716. doi: 10.1038/srep33716 (PMC5028767; doi:10.1038/srep33716)
Supplement: Supplementary Information [file srep33716-s1.pdf]

Candida albicans Dbf4-dependent Cdc7 kinase plays a novel role in the inhibition of hyphal development

Wei-Chung Lai[1], Tschen-wei Chang[1§], Chang Hao Wu[1§], Shu-Ya Yang[1], Tai-Lin Lee[2], Wan Chen Li[1,3], Ting Chien[1,4], Yu-Che Cheng[1,4], and Jia-Ching Shieh[1,5] *

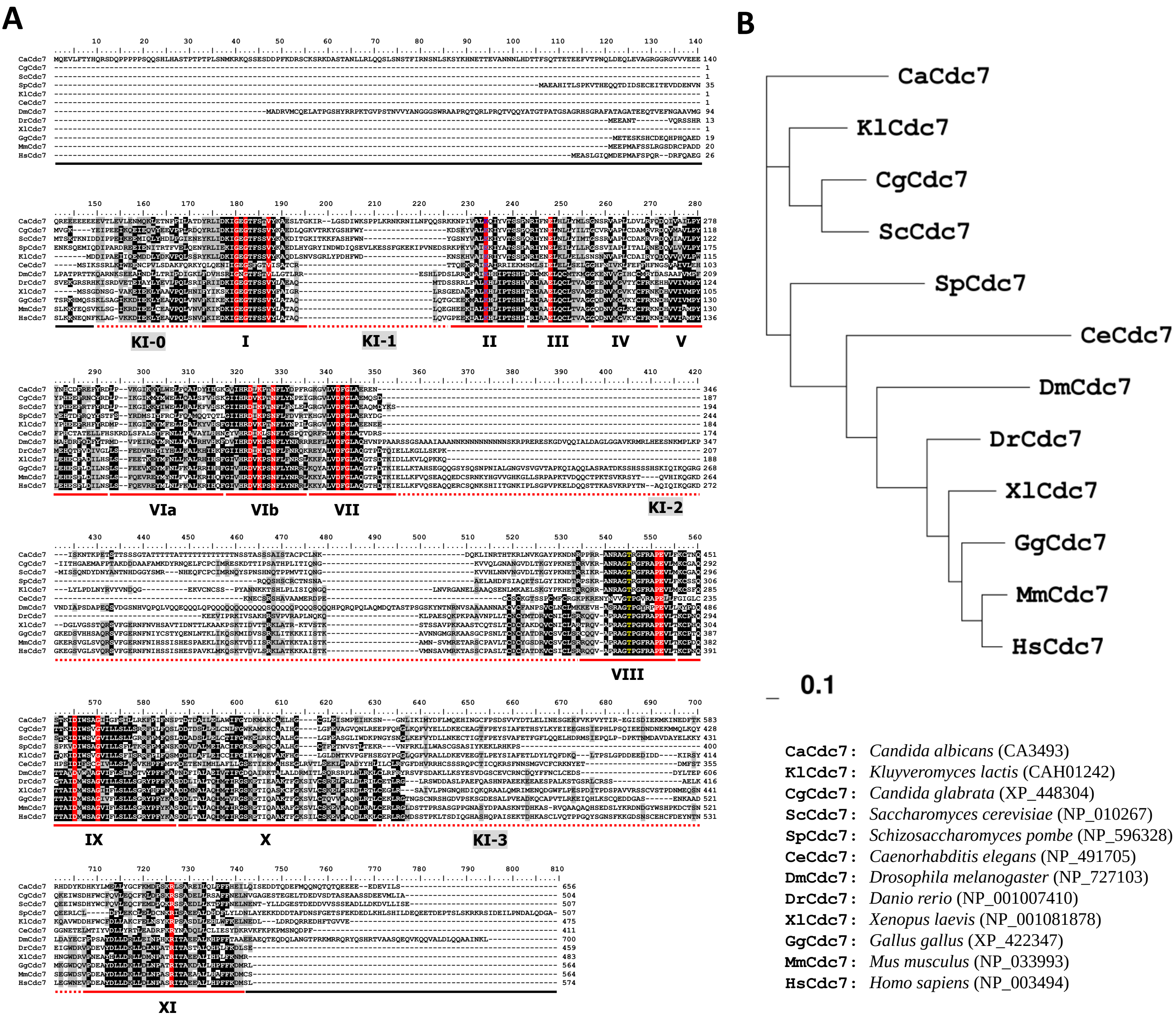

Figure S1. A. Alignment of Cdc7 protein sequences by ClustalW program[1,2]. The alignment revealed that *Ca*Cdc7 encodes a serine/threonine kinase[3] with homology both in sequence and organization to Cdc7 across evolutionary spectrum. *Ca*Cdc7 possesses a lysine 232 (in blue) for ATP binding that is essential for the catalytic activity. *Ca*Cdc7 also has a threonine 437 (in yellow), a phosphoacceptor residue that is required for

the kinase activation. Particularly, *Ca*Cdc7 possesses Cdc7 characteristic insertions of KI-0, KI-1, KI-2, and KI-3. *Ca*Cdc7 also has specific regions located at N- and C-terminus. The C-terminal tail of Cdc7 is known to be essential for interacting with Dbf4[4]. Together with KI-2 and KI-3, such an interaction becomes efficient[5]. The less conserved C-terminal tail may be critical for species-specific interaction with the Dbf4. The serine/threonine kinase specific subdomains are underlined in red and indicated by Roman numerals below the sequences. The Cdc7-specific insertions are shown dash-lined in red and indicated by KI-0~3 with shading under the sequences. The identical residues are shaded in black; the conserved changes are shaded in grey; the highly conserved invariant residues within the subdomains are shaded in red. The *C. albicans* specific regions are underlined in black. **B.** Phylogenetic analysis Neighbor-Joining/UPGMA method version 3.6a2.1 and drawn by the PHYLIP program[6] showed that *Ca*Cdc7 is clustered together to its fugal counterparts and that *Ca*Cdc7 is related to *S. cerevisiae*; hence the function of *Ca*Cdc7 might be referred to the well-studied *Sc*Cdc7 by analogy. Bar represents 0.1 nucleotide substitutions per site. Species origin of each Cdc7 (with the accession number of genome database) used in the analysis is shown

References for Figure S1

1 Larkin, M. A. *et al.* Clustal W and Clustal X version 2.0. *Bioinformatics* **23**, 2947-2948; DOI: 10.1093/bioinformatics/btm404 (2007).

2 Thompson, J. D., Higgins, D. G. & Gibson, T. J. CLUSTAL W: improving the sensitivity of progressive multiple sequence alignment through sequence weighting, position-specific gap penalties and weight matrix choice. *Nucleic Acids Res* **22**, 4673-4680 (1994).

3 Hanks, S. K. & Quinn, A. M. Protein kinase catalytic domain sequence database: identification of conserved features of primary structure and classification of family members. *Methods Enzymol* **200**, 38-62 (1991).

4 Patterson, M., Sclafani, R. A., Fangman, W. L. & Rosamond, J. Molecular characterization of cell cycle gene CDC7 from Saccharomyces cerevisiae. *Mol Cell Biol* **6**, 1590-1598 (1986).

5 Sato, N., Arai, K. & Masai, H. Human and Xenopus cDNAs encoding budding yeast Cdc7-related kinases: in vitro phosphorylation of MCM subunits by a putative human homologue of Cdc7. *Embo J* **16**, 4340-4351 (1997).

6 Retief, J. D. Phylogenetic analysis using PHYLIP. *Methods Mol Biol* **132**, 243-258 (2000).

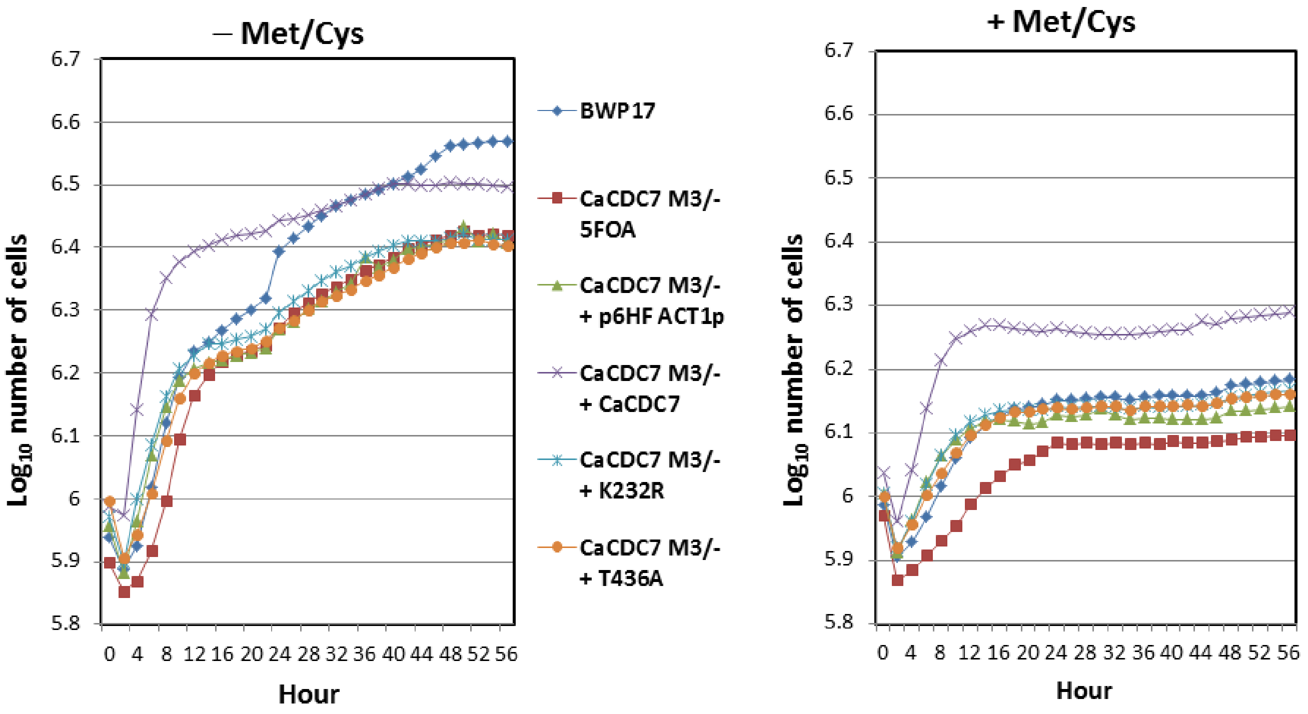

Figure S2. *Ca*Cdc7 is crucial for the growth of *C. albicans*. Cells of strain *CaCDC7* M3/– were transformed with either the empty p6HF-*ACT1*p or p6HF-*ACT1*p-*Ca*CDC7, capable of constitutively expressing wild-type *Ca*Cdc7 (*CaCDC7*), the catalytically inactive *Ca*Cdc7 (K232R), or the phosphoacceptor-deficient *Ca*Cdc7 (T437A). Cells of each of the strains, together with the BWP17 from which the *CaCDC7* M3/– was derived, were grown in the SD medium with required supplements in the presence (+ Met/Cys) or absence (–Met/Cys) of 2.5 mM methionine and cysteine at 30°C for 56 h. The cultures of each of the strains were collected every 2 h and subjected to determine the absorbent at OD_{600}. The growth curves are presented as the Log_{10} number of cells per ml.

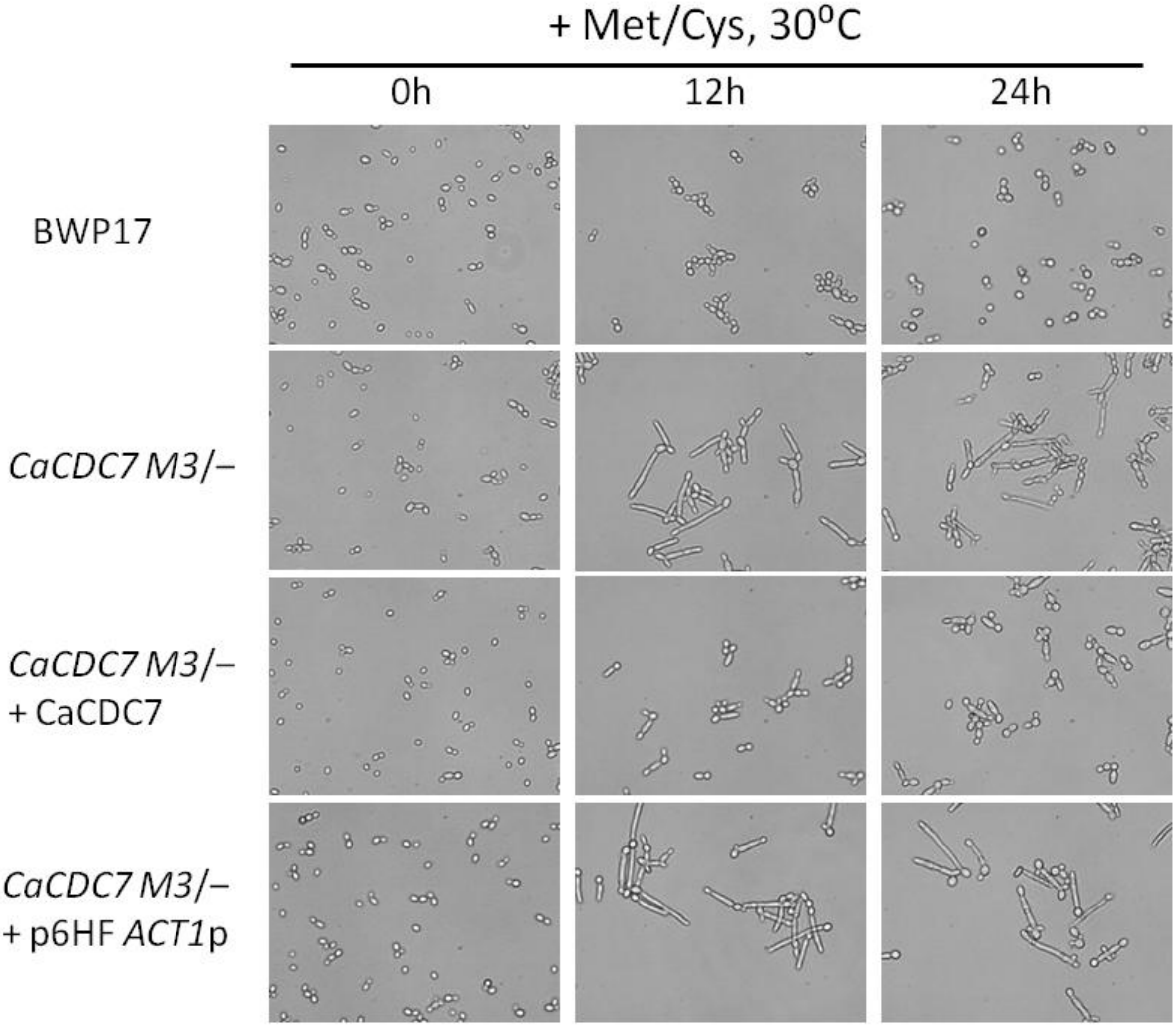

Figure S3. *Ca*Cdc7 is responsible for the inhibition of hyphal growth in *C. albicans*. Cells of strain *CaCDC7* M3/– carrying either the empty p6HF-*ACT1*p or p6HF-*ACT1*p-*Ca*C*DC7*, capable of constitutively expressing wild-type *Ca*Cdc7 (*CaCDC7*), together with BWP17, were grown in the SD medium with required supplements in the presence (+ Met/Cys) of 2.5 mM methionine and cysteine at 30°C for the indicated time. The cultures were subjected to microscopic assessment.

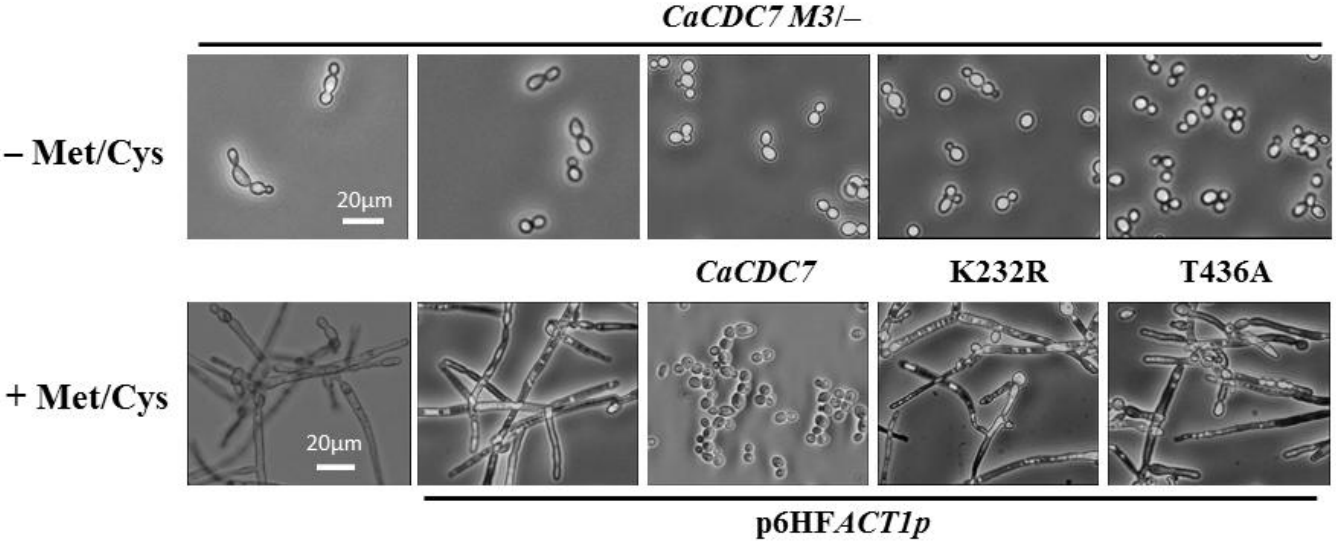

Figure S4. *Ca*Cdc7 is important for maintenance of the hyphal development in *C. albicans*. Cells of strain *CaCDC7* M3/– carrying either the empty p6HF-*ACT1*p, p6HF-*ACT1*p-*Ca*C*DC7,* capable of constitutively expressing wild-type *Ca*Cdc7 (*CaCDC7*), the catalytically inactive *Ca*Cdc7 (K232R), or the phosphoacceptor-deficient *Ca*Cdc7 (T437A) were grown in the SD medium with required supplements in the presence (+ Met/Cys) or absence (–Met/Cys) of 2.5 mM methionine and cysteine at 30°C for 32 h. The cultures were subjected to microscopic assessment. Bars represent 20 μm.

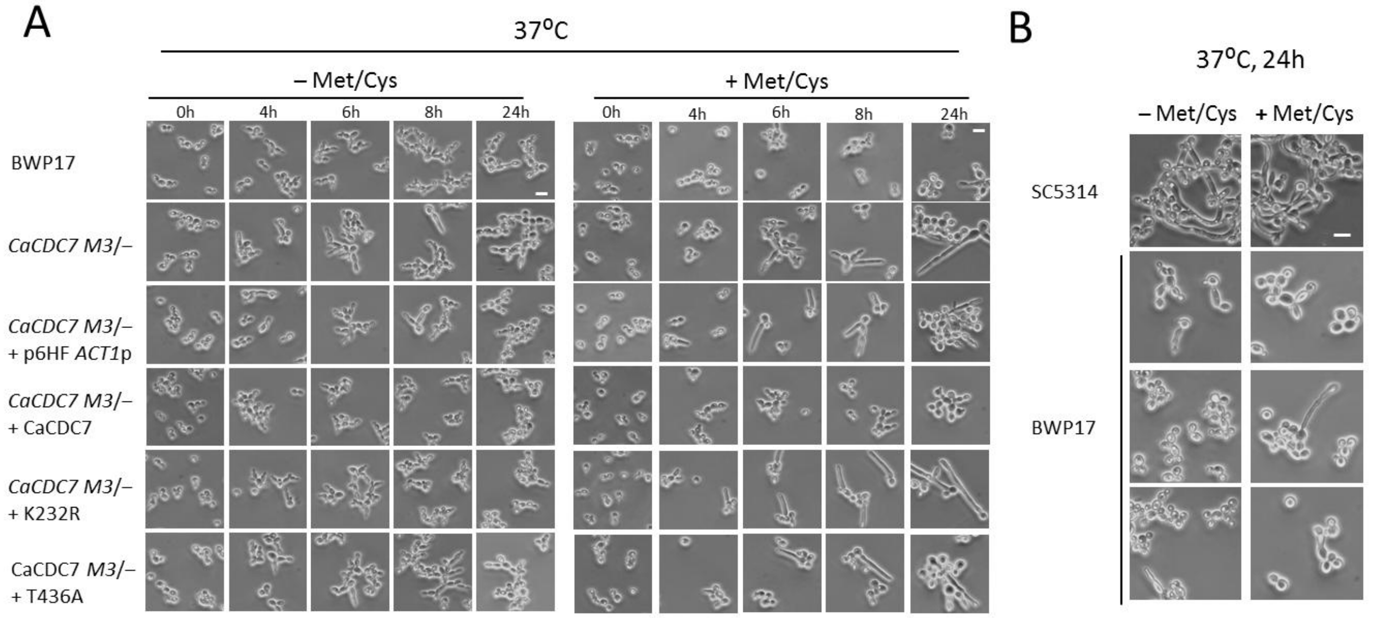

Figure S5. *Ca*Cdc7 is important for the initiation of hyphal growth in *C. albicans*. **A.** Cells of each of the strains same as in **Figure S2** and BWP17 were grown in the SD medium with required supplements in the presence (+ Met/Cys) or absence (–Met/Cys) of 2.5 mM methionine and cysteine for the indicated time at 37°C prior to the microscopic assessment. **B.** Cells of the wild-type strain SC5314 and the auxotrophic strain BWP17 were grown in the same condition and collected at 24 h for the microscopic assessment. Bars represent 10 μm.

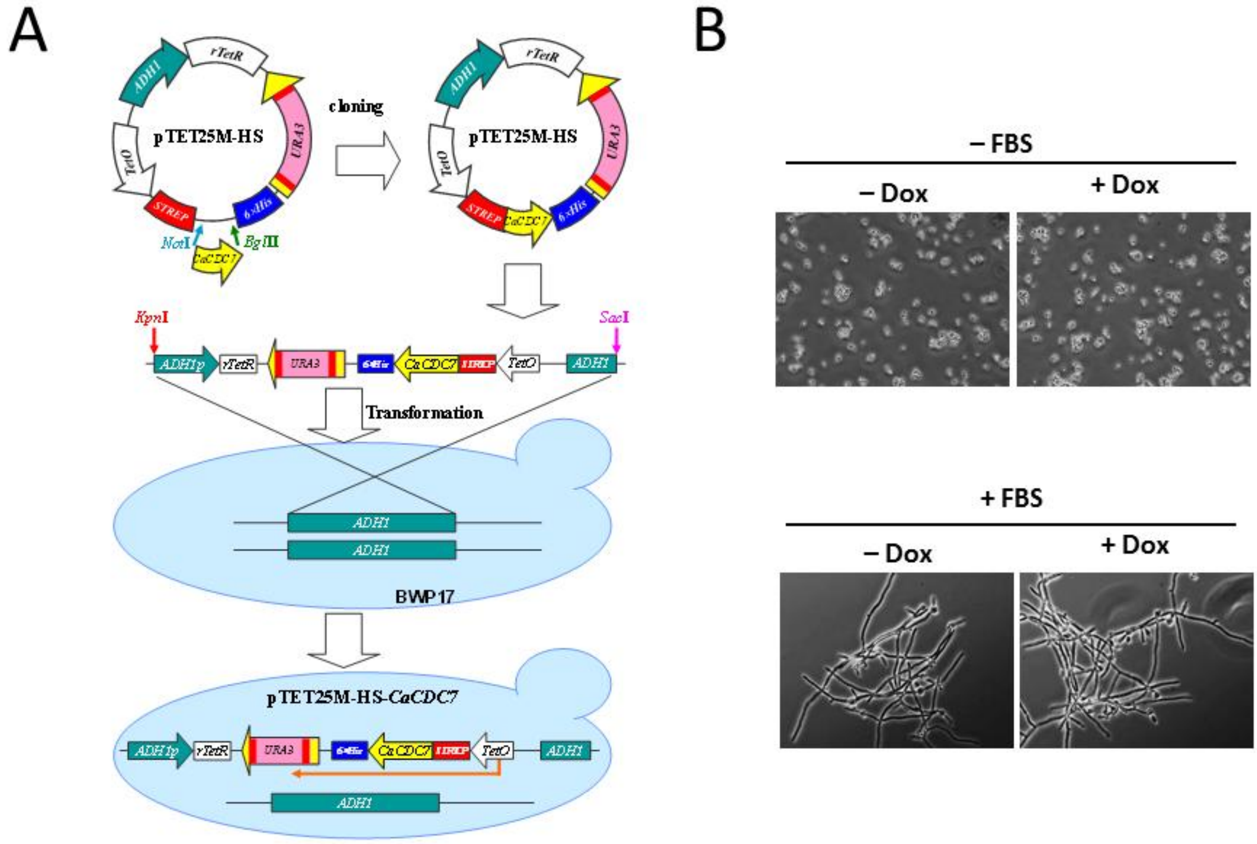

Figure S6. *C. albicans* cells overexpressing *CaCDC7* could not reverse the hyphal development under hyphae-inducing condition. **A.** The coding region of *CaCDC7* was cloned into the vector pTET25M-HS. The recombinant plasmid was linearized before transforming into *C. albicans* strain BWP17 for *URA3*$^{+}$ prototrophy, which is capable of inducing *CaCDC7* expression in the presence of doxycycline (Dox). **B.** Cells of this strain were grown in YPD in the presence (+ Dox) or absence (–Dox) of 40 mM doxycycline with (+FBS) or without (–FBS) 10% fetal bovine serum (FBS) at 30°C for 3 h.
